# Development and Applications of PLGA Hydrogels for Sustained Delivery of Therapeutic Agents

**DOI:** 10.3390/gels10080497

**Published:** 2024-07-26

**Authors:** Anita Ioana Visan, Irina Negut

**Affiliations:** National Institute for Lasers, Plasma and Radiation Physics, 409 Atomistilor Street, 077125 Magurele, Romania; anita.visan@inflpr.ro

**Keywords:** poly(lactic-co-glycolic acid) (PLGA), hydrogels, sustained delivery, therapeutic agents

## Abstract

Poly(lactic-co-glycolic acid) (PLGA) hydrogels are highly utilized in biomedical research due to their biocompatibility, biodegradability, and other versatile properties. This review comprehensively explores their synthesis, properties, sustained release mechanisms, and applications in drug delivery. The introduction underscores the significance of PLGA hydrogels in addressing challenges like short half-lives and systemic toxicity in conventional drug formulations. Synthesis methods, including emulsion solvent evaporation, solvent casting, electrospinning, thermal gelation, and photopolymerization, are described in detail and their role in tailoring hydrogel properties for specific applications is highlighted. Sustained release mechanisms—such as diffusion-controlled, degradation-controlled, swelling-controlled, and combined systems—are analyzed alongside key kinetic models (zero-order, first-order, Higuchi, and Peppas models) for designing controlled drug delivery systems. Applications of PLGA hydrogels in drug delivery are discussed, highlighting their effectiveness in localized and sustained chemotherapy for cancer, as well as in the delivery of antibiotics and antimicrobials to combat infections. Challenges and future prospects in PLGA hydrogel research are discussed, with a focus on improving drug loading efficiency, improving release control mechanisms, and promoting clinical translation. In summary, PLGA hydrogels provide a promising platform for the sustained delivery of therapeutic agents and meet diverse biomedical requirements. Future advancements in materials science and biomedical engineering are anticipated to further optimize their efficacy and applicability in clinical settings. This review consolidates the current understanding and outlines future research directions for PLGA hydrogels, emphasizing their potential to revolutionize therapeutic delivery and improve patient outcomes.

## 1. Introduction

With the exponential growth of scientific knowledge, the field of poly(l-lactide-co-glycolide) (PLGA) hydrogels has witnessed remarkable progress. This study aims to explore details of the synthesis techniques employed in creating PLGA hydrogels, highlighting factors that govern the formation process. Furthermore, it meticulously examines the special properties exhibited by these hydrogels, considering their biodegradability, biocompatibility, and tunable mechanical characteristics. The applications of PLGA hydrogels in the biomedical domain are truly remarkable. Their versatility and ability to encapsulate and deliver therapeutic agents have garnered significant attention. By expanding the horizons of drug delivery systems, tissue engineering, regenerative medicine, and wound healing, PLGA hydrogels exhibit immense promise. Continuous research in this field furthers our understanding and fosters the development of innovative strategies, pushing the boundaries of medical technology. Therefore, this detailed extended review serves as an invaluable resource for researchers, scientists, and medical practitioners, providing comprehensive insights into the advancements, potential challenges, and exciting prospects of PLGA hydrogels in the field of biomedical applications [1]. Over the years, hydrogels have continued to evolve, as no single material can meet the growing technological demand. Various synthesis techniques have been developed to design smart hydrogels suitable for diverse applications, enabling the fabrication and modification of the structural, mechanical, and sensitive properties of these outstanding materials. Smart hydrogels are fascinating materials between elastomers and gels that are characterized by their ability to swell and shrink in response to environmental stimuli such as pH, temperature, light, ions, enzymes, electric or magnetic fields, and biomolecules. This property has led to a growing interest in using hydrogels for various applications in the fields of drug delivery release, wound healing, tissue engineering, and regenerative medicine. At the same time, synthetic advances have created a new generation of hydrogels, ensuring the applicability of smart, responsive materials in the most complex physiological environments, inspired by nature’s small building units [2,3,4,5].

**Figure 1:**
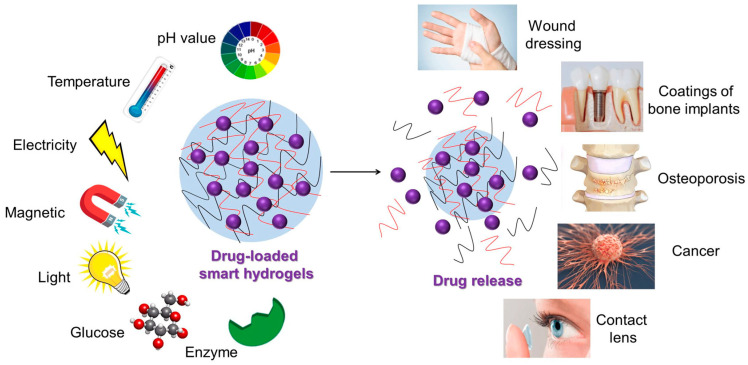
Illustration of various external stimuli, including pH, temperature, electricity, magnetics, light, and biomolecules (including glucose and enzymes), which are controlling the drug release from a smart hydrogel, and their biomedical applications [6].

Figure 1 illustrates the various external stimuli—such as pH, temperature, electricity, magnetics, light, and biomolecules (including glucose and enzymes)—that can control drug release from a smart hydrogel, highlighting their potential biomedical applications.

The topic of composite hydrogels for sustained release of therapeutic agents has been extensively reviewed in the literature. For instance, Liu, Nakamura, and Lowman (2003) conducted a comprehensive review identifying the potential of composite hydrogels in providing controlled and sustained release of therapeutic agents, highlighting their ability to enhance drug stability and bioavailability [7]. Similarly, Liu et al. (2023) explored the application and development of hydrogel biomaterials for the treatment of intervertebral disc degeneration, concluding that hydrogels can effectively mimic the natural extracellular matrix, providing mechanical support and promoting tissue regeneration [8].

Despite these valuable contributions, there remain significant gaps, particularly in the areas of long-term controlled release and the development of hydrogels with enhanced mechanical properties. For instance, Cisneros et al. (2021) discussed the long-term controlled release of simvastatin from photoprinted triple-networked hydrogels, composed of modified chitosan and PLA–PEG micelles, yet there is still a need for further research into the scalability and clinical applicability of these systems [9]. Additionally, Alsaab et al. (2022) reviewed the advancements in PLGA-based nanomedicine, underscoring the importance of developing nanocomposite hydrogels for improved therapeutic efficacy and targeting [10].

These gaps are crucial because they highlight the need for more robust and versatile hydrogel systems that can be tailored to specific therapeutic needs. Therefore, by building on the foundational work of previous reviews, this review seeks to advance the application of hydrogel biomaterials in drug delivery, ultimately contributing to the identification of more effective therapeutic strategies.

The need to review the development and application of PLGA hydrogels for the sustained delivery of therapeutic agents arises from the growing importance of this biodegradable polymer in the field of drug delivery systems. PLGA hydrogels provide a versatile and efficient platform for the controlled release of a variety of therapeutic agents, including small molecules, proteins, and nucleic acids. Their biocompatibility, adjustable degradation rates, and ability to encapsulate different types of drugs make them ideal for various medical applications. This review not only summarizes the latest advances and innovations in PLGA hydrogel formulations but also highlights their clinical implications and addresses the current challenges and future directions. This provides researchers and healthcare professionals with a valuable resource to advance further research and development and ultimately improve patient outcomes through more effective and targeted therapies.

### Definition and Composition of PLGA Hydrogels

PLGA is a biodegradable and biocompatible polymer widely used in biomedical applications, particularly in drug delivery systems, tissue engineering, and regenerative medicine. The hydrogel form of PLGA combines the advantageous properties of hydrogels—such as its high water content and tunable mechanical properties—with the favorable characteristics of PLGA, including its degradability and versatility in drug release profiles [11].

PLGA is a copolymer of lactic acid and glycolic acid. When combined, they form a hydrogel that can absorb and retain water. The ratio of lactic acid to glycolic acid affects the hydrogel’s properties and degradation rate. Common ratios are 50:50, 75:25, or 25:75, each suitable for different applications [12]. 

PLGA’s structural formula comprises repeating units of lactic acid and glycolic acid, which degrade into biocompatible and metabolizable by-products. 

**Figure 2:**
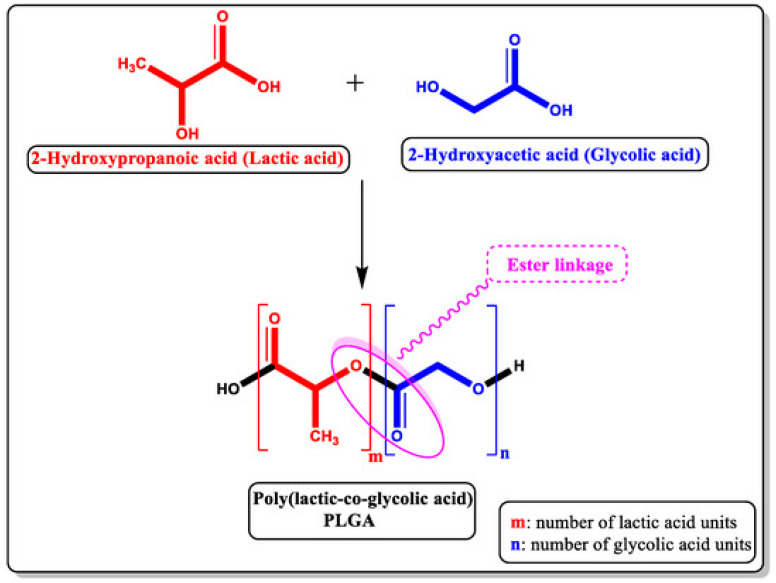
The structural formula of PLGA [10].

The general structural formula of PLGA (Figure 2) can be written as follows:

–[O–CH(CH_3_)–CO]_m_–[O–CH_2_–CO]_n_–– (1)

where

- m represents the number of lactic acid units.
- n represents the number of glycolic acid units.

The ratio of m to n can vary depending on the specific type of PLGA used. For example, PLGA with a 50:50 ratio of lactic acid to glycolic acid would have equal numbers of m and n. The structure can be visualized as alternating chains of the lactic acid and glycolic acid units. The ratio of these units determines the polymer’s physical and chemical properties, such as its degradation rate and mechanical strength [13].

To form a hydrogel, PLGA polymers are crosslinked, which can be achieved through various chemical or physical methods. 

Chemical crosslinking involves covalent bonding, often using agents such as glutaraldehyde, carbodiimides, or photoinitiators that activate under UV light [14]. 

Physical crosslinking can involve processes such as ionic interactions or crystallization. As a hydrogel, PLGA networks incorporate water into their structure, significantly influencing their mechanical properties and biological interactions [15]. The water content allows the hydrogel to swell, providing a moist environment conducive to cellular activities and facilitating the controlled release of therapeutic agents. To enhance the functionality of PLGA hydrogels, various additives can be included. These may include bioactive molecules like growth factors or drugs, nanoparticles for targeted delivery, or other polymers to modify mechanical properties or degradation rates [16]. Fillers such as hydroxyapatite or bioactive glass can be added to improve bioactivity, especially for bone tissue engineering applications [17].

**Figure 3:**
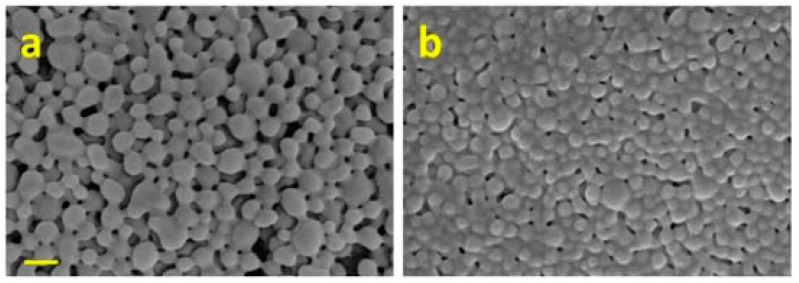
Scanning electron microscopy (SEM) images of (**a**) poly(lactic-co-glycolic) acid (PLGA) nanoparticles (P NPs) and PLGA–poloxamer nanoparticles (PP NPs); (**b**) PLGA–poloxamer nanoparticles (PP NPs). The scale bar is 200 nm [18].

SEM micrographs play a pivotal role in the development and characterization of PLGA-based biomedical applications. They provide critical insights into the morphology, size, and surface properties of PLGA particles which are essential for optimizing their performance in drug delivery systems and tissue engineering scaffolds. The detailed visualization afforded by SEM ensures that PLGA-based systems are designed with the precision necessary for effective clinical outcomes. A study by Silvestri et al. [18] explored the effects of surface hydrophilicity on PLGA–poloxamer nanoparticles in an in vivo animal model. SEM micrographs revealed significant details about the surface texture and porosity of the PLGA hydrogels. Hydrogels with varying degrees of hydrophilicity were synthesized, and SEM imaging demonstrated how these variations impacted the surface morphology. Hydrogels with higher hydrophilicity showed more porous structures, which can facilitate better cell infiltration and nutrient diffusion, enhancing their suitability for biomedical applications (Figure 3). 

In another study [19], the synthesis and characterization of PLGA nanoparticle/4-arm-PEG hybrid hydrogels with controlled porous structures were reported. SEM micrographs from this study provided a comprehensive view of the internal and external morphology of the hydrogels. The hybrid hydrogels displayed a well-defined porous network, which is essential for drug delivery systems as it influences the release rate of encapsulated drugs. The controlled porosity observed in the SEM images indicates the effectiveness of the synthesis method in producing hydrogels with specific structural characteristics can be found in Ref. [19].

These micrographs are essential in understanding the physical attributes of PLGA-based delivery systems which directly influence their performance in biomedical applications.

SEM images typically show that PLGA particles exhibit a spherical shape with a smooth surface. The size of these particles can vary significantly based on the preparation method. For instance, Wang et al. demonstrated that the particle size distribution of PLGA nanoparticles could be controlled to enhance the efficacy of localized chemotherapy for tumors [20]. Similarly, Hsu et al. highlighted the importance of particle size in the development of nanoparticle–hydrogel composites for ocular drug delivery, showing that SEM micrographs helped confirm the uniformity and desirable size of the PLGA nanoparticles used [21].

The surface texture observed in SEM micrographs can provide insights into the encapsulation efficiency and drug release profiles. For example, a study by Wang et al. showed that the surface of PLGA nanoparticles loaded with sorafenib was smooth, indicating efficient drug encapsulation within the particles [22]. Similarly, Kong et al. utilized SEM to confirm the smooth texture of PLGA-based hydrogels, which is crucial for controlled drug release during in situ therapies [23].

PLGA’s versatility in drug delivery is well-documented across various studies. The incorporation of PLGA nanoparticles into hydrogels can create composite systems with enhanced drug release properties. For instance, Folle et al. used SEM to analyze the integration of thyme-oil-loaded PLGA nanoparticles into a hydrogel matrix, designed for treating skin inflammation. The micrographs confirmed the successful embedding of nanoparticles within the hydrogel, crucial for ensuring the sustained release of the active ingredient [24].

The unique combination of properties makes PLGA hydrogel an excellent candidate for a wide range of biomedical applications. In drug delivery, its degradability allows for the controlled release of drugs over a sustained period. In tissue engineering, its biocompatibility and ability to mimic the extracellular matrix environment support cell proliferation and differentiation [16]. Furthermore, the flexibility in its composition enables customization to fulfill particular medical requirements. While PLGA is generally considered safe and biocompatible, understanding its degradation behavior and potential for localized toxicity is important for its application in biomedical devices. Proper design and formulation can mitigate adverse effects, ensuring PLGA remains a valuable material in the medical and pharmaceutical fields [10].

PLGA is generally considered biocompatible. When implanted in the body, it degrades into its monomers, lactic acid and glycolic acid, which are naturally metabolized and eliminated by the body. Lactic acid enters the tricarboxylic acid (TCA) cycle and is metabolized to carbon dioxide and water, while glycolic acid is either excreted through the kidneys or metabolized into glycine, which enters the TCA cycle. The degradation of PLGA results in the production of lactic acid and glycolic acid. While both are naturally occurring substances in the body, an excessive accumulation can lead to localized pH changes and inflammation. This can be particularly problematic if large amounts of PLGA are used, leading to acidic microenvironments which can affect cell viability and function [25]. Excessive acidity from PLGA degradation can cause a range of cellular responses. These may include apoptosis or necrosis if the local environment becomes too acidic, affecting the surrounding tissues. Additionally, an increase in the concentration of glycolic acid can cause metabolic acidosis if the body’s buffering capacity is overwhelmed, although this is rare and typically only occurs with very high doses [26].

The immune response to PLGA is typically minimal, but it can vary depending on the specific formulation, molecular weight, and degradation rate of the polymer. In some cases, the body may recognize PLGA as a foreign material, leading to a mild inflammatory response. This is usually transient and resolves as the polymer degrades and is cleared from the body [25]. Macrophages and other immune cells may infiltrate the area where PLGA is degrading, which can sometimes lead to fibrous encapsulation, a common response to foreign materials. However, this reaction is generally mild and can be managed through the careful design and placement of PLGA-based devices [27].

Long-term studies on the use of PLGA in humans have generally shown it to be safe. However, prolonged exposure to the degradation products can potentially cause issues in sensitive tissues or organs. Therefore, the design of PLGA-based devices often includes considerations to control the degradation rate and ensure the safe elimination of by-products [13].

For instance, devices that slowly degrade over time are less likely to cause a sudden spike in lactic and glycolic acid concentrations, reducing the risk of localized toxicity. Additionally, the molecular weight and copolymer ratio can be adjusted to tailor the degradation rate to match the intended use [25].

To conclude, PLGA hydrogel represents a sophisticated material combining the advantageous properties of both PLGA polymers and hydrogels. Its tunable composition and favorable characteristics make it a powerful tool in advancing medical science and improving patient care through innovative drug delivery systems and tissue engineering solutions [28].

## 2. Synthesis Methods of PLGA Hydrogels

In the following section, we will summarize several common syntheses approaches for drug-loaded PLGA hydrogels. The state-of-the-art synthesis strategies and the corresponding properties of the PLGA hydrogel are also presented. In recently published studies, great effort has been devoted to optimizing the properties to fit the specific requirements in different applications, and the related knowledge is summarized.

To predominate therapeutic applications, PLGA hydrogels, as well as many other hydrogels, should have a uniform shape for further research and subsequent transition injectable properties to be used for minimally invasive local therapy, especially those administered via minimally invasive techniques, e.g., syringe injection. Therefore, the following synthesis methods are good enough in the two stated perspectives and are generally considered easily controllable and reproducible methods for PLGA hydrogel and need no or fewer harmful chemical reagents [29]. 

The properties, notably the temperature, pH, and solvent, associated with biomedical applications can be easily controlled to fit the various clinical applications using traditional synthesis methods; for example, a PLGA solution in an acid organic solvent can be easily prepared by adjusting the solvent component before injection. After the hydrogel is formed, the temperature of the body plays the most important role in the sol-gel transition process [30]. 

**Figure 4:**
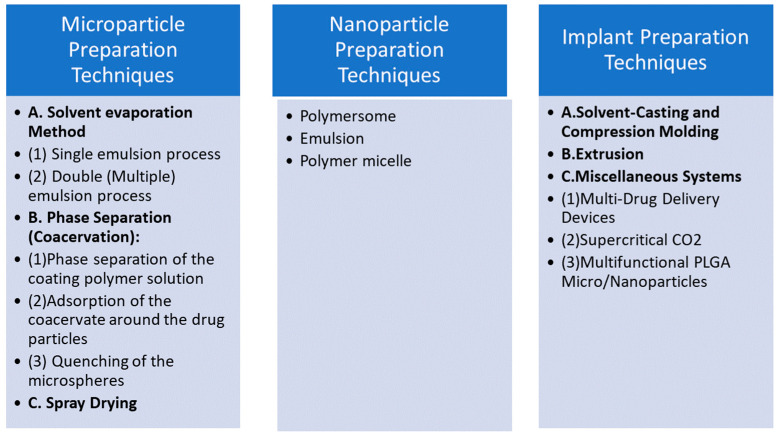
The figure shows the different and main fabrication techniques for drug-loaded PLGA hydrogels and the factors related to their degradation besides their characteristics, responsiveness, advantages, and disadvantages [10].

Figure 4 provides an overview of the different fabrication techniques for PLGA for drug-loaded PLGA hydrogels, highlighting factors related to its degradation, characteristics, responsiveness, and advantages and disadvantages. Given its biodegradability and biocompatibility, PLGA is extensively utilized as a hydrophobic polymer to create various carriers, such as polymersomes, emulsions, polymeric micelles, and more, making it a versatile choice in drug delivery systems.

PLGA exhibits both hydrophilic and hydrophobic characteristics, which depend on its copolymer composition and the ratio of lactic acid to glycolic acid. Generally, the glycolic acid units are more hydrophilic, while the lactic acid units are more hydrophobic due to the presence of the methyl group. Consequently, the overall hydrophilicity or hydrophobicity of PLGA can be tailored by adjusting the lactic to glycolic acid ratio. For instance, a higher content of glycolic acid increases the hydrophilicity, whereas a higher content of lactic acid enhances the hydrophobicity. When PLGA has a higher ratio of lactic acid, it tends to be more hydrophobic. The methyl groups in the lactic acid units repel water, reducing the polymer’s affinity for water. Increasing the glycolic acid content makes the polymer slightly less hydrophobic. Glycolic acid lacks the hydrophobic methyl group, making it more polar than lactic acid. Consequently, PLGA with a higher glycolic acid content has better water affinity, although it remains predominantly hydrophobic [31].

PLGA hydrogel synthesis is mainly done through physical or chemical crosslinking methods. The physical crosslinking method relies on the chemical modification of a PLGA polymer, e.g., thiol modification of the PLGA end group, to reduce the critical association concentration. The chemical crosslinking method relies on the introduction of a third component to react with the PLGA polymer more or less. In general, the chemical crosslinking method achieves a tighter network structure and higher modulus than the physical one, due to the generation of additional crosslink sites by introducing a third component [32]. 

### 2.1. Chemical Crosslinking

The role of crosslinking in the preparation of PLGA hydrogels is to hold the polymer in a 3D structure. When prepared as hydrogels, hydrophilic polymer chains are propagated in the preparation system. Without crosslinking, these chains would dissolve when heated or stirred. Permanent polymer crosslinking holds the network in place and prevents dissolution. Crosslinking within the polymer network can occur with or without external crosslinking agents. External crosslinking often involves covalent bonds between the polymer chains, requiring the presence of functional groups on the polymer chain. However, monomer or oligomer compounds without external crosslinking effects cannot form covalent bonds [33]. 

For the external charging method, the presence of functional groups is essential as they can serve as binding sites between the polymer chains. If the appropriate concentration of functional groups is present in the polymer, crosslinking can be achieved by adjusting the concentration of monomers or oligomers. The addition of external energy facilitates the formation of covalent bonds, resulting in the final curing of the polymer network structure. On the other hand, due to the dual-phase nature of PLGA hydrogels, with hydrophobicity in the central region and hydrophilicity in the head and tail, ammonolysis or aminolysis reactions are typically used to achieve crosslinking. In this article, we summarize the preparation method, properties, and biomedical applications of chemically crosslinked PLGA hydrogels. Additionally, we provide future trends for PLGA hydrogels in the concluding section [34].

### 2.2. Physical Crosslinking

Techniques based on the physical interaction of polymer chains do not destroy the polymer microstructure and maintain the sol-gel transition properties. Many of the desirable features of chemical hydrogel crosslinking can also be approached through proper physical hydrogel formation. The major advantage of this class of hydrogels is the lack of potential for cytotoxic residue or the need to use cytotoxically controlled chemicals or irradiation sources. Thus, these hydrogels are very useful when interacting with living systems [35].

H-bond crosslink hydrogels are derived from polymers possessing H-bonding sites. These sites form intermolecular or intramolecular H-bonded secondary structures. Although H-bonding-based hydrogels are mostly derived from self-associative polymers, H-bonding sites can also be incorporated into synthetic polymers in a bioinspired manner. In these cases, the H-bonded sites are built into the hydrogel by conjugating natural, amino acid-derived, and/or peptidic moieties into the polymer chain. Such mutations create nanostructures that can form H-bonds amongst each other. In the case of self-associative polymers, no external driving force is needed for gelation [2].

### 2.3. Fabrication Techniques for PLGA Hydrogels

The development of hydrogels as delivery platforms for therapeutic agents, for example, drugs, peptides, proteins, and growth factors, has received more attention due to their unique hydration and physical properties. Conventional hydrogels are prepared with physical or chemical crosslinking and, in most cases, lack loading capacity for hydrophobic molecules. These hydrogels, derived from compressed PLGA, exhibit fascinating phase behavior near the glass transition temperature (Tg). They are fabricated through casting/quenching processing and applied for the sustained delivery of various drugs at the same time, feasible for model drugs with different hydrophilicities [36]. Different drugs can achieve different sustained release profiles through these PLGA hydrogels, suggesting they are versatile for the delivery of many therapeutic agents. The phase transition temperature of the hydrogel is potentially used as a guideline for sustained release (burst or slow release) of the drugs [37]. The ability to control the release rate of drugs is a critical aspect in modern pharmaceutics. Hydrogel-based delivery systems have garnered considerable attention due to their unique properties, including their biocompatibility, excellent mechanical strength, and tunable release profiles. Notably, hydrogels have emerged as promising carriers for a diverse range of therapeutic agents, including small drug molecules, peptides, proteins, and growth factors [38]. One key advantage of PLGA hydrogels is their ability to facilitate sustained drug delivery. Regardless of the hydrophilicity of the model drug, these hydrogels have demonstrated immense versatility in accommodating a wide variety of therapeutic agents. Consequently, different drugs have exhibited distinct sustained release profiles within the PLGA hydrogel matrix. This observation underscores the potential of PLGA hydrogels as a promising platform for efficient drug delivery [39]. An interesting perspective is to use the Tg of the PLGA hydrogel as a crucial guideline for regulating sustained release mechanisms. By carefully manipulating temperature, the desired drug release kinetics can be achieved, be it burst release or slower, controlled release over a longer period of time. This temperature-dependent control of drug release holds promise for tailoring hydrogel-based delivery systems to specific therapeutic needs [40]. The synthesis of PLGA hydrogels specifically for sustained drug delivery involves several methods designed to optimize the encapsulation, release profiles, and biocompatibility of therapeutic agents [41]. Emulsion solvent evaporation involves dissolving PLGA and the drug in an organic solvent, followed by emulsification in an aqueous phase. The organic solvent is then evaporated, leading to the formation of drug-loaded PLGA nanoparticles or microspheres that can be crosslinked to form hydrogels, ensuring sustained release of the drug [31]. 

Solvent casting/particulate leaching implies that the PLGA is dissolved in a solvent along with the drug, cast into a mold containing a leachable particulate (e.g., salt or sugar), and then the solvent is evaporated. The particles are then released to create a porous hydrogel structure that enables sustained drug release [42]. 

In the case of the electrospinning technique, PLGA and the drug are dissolved in a solvent, and the solution is electrospun to create fine fibers. These fibers are then collected and crosslinked to form a hydrogel matrix. The porous structure of the electrospun fibers facilitates controlled and sustained drug release. Solvent casting employs water-soluble particulates like salt or sugar, which are leached out to create porous structures, reducing environmental impact [43]. 

Another synthesis technique is thermal gelation. PLGA copolymers are designed to undergo gelation at specific temperatures. The drug is mixed with the PLGA solution, which then forms a hydrogel upon a temperature change. This method allows the hydrogel to encapsulate the drug efficiently and release it in a controlled manner. Thermal gelation does not require organic solvents, thus reducing the potential toxicological impacts [44]. 

In the photopolymerization method, PLGA can be functionalized with photoreactive groups, and the active ingredient can be mixed into this solution. When exposed to light, crosslinking occurs, forming a hydrogel. Photopolymerization provides precise control over the gelation process and drug distribution within the hydrogel. Moreover, this method eliminates the need for chemical crosslinkers and reduces the generation of hazardous waste [45].

Table 1 provides an overview of different synthesis methods for PLGA hydrogels, highlighting their advantages and disadvantages and providing case examples.

### 2.4. Emulsion Solvent Evaporation Technique

The emulsion technique for PLGA hydrogel fabrication involves the careful selection of solvents, emulsification of PLGA, hardening of emulsion droplets, and formation of porous structures [31]. Typically, to prepare PLGA hydrogels using the emulsion technique, a porogen or polymer blend is prepared with a hydrogel solvent, followed by PLGA dissolution in the polymer. Solvents are carefully chosen such that they can effectively dissolve PLGA and porogens, ensuring a thorough mixture. In addition, these solvents must be partially miscible with each other to enable controlled phase separation of these two polymers. This controlled phase separation is crucial in achieving the desired structure and properties of the hydrogel [50]. Moreover, it is essential for the chosen solvents to be non-toxic and low-boiling. This enables easy removal of the solvents at biological temperatures, preventing any potential harm to the surrounding environment or biological systems. Therefore, the selection of suitable solvents is a critical step in the fabrication process [50].

To promote emulsion formation, an emulsifying agent is then dissolved in the solvent or polymer mixture. This emulsifying agent aids in achieving a stable and uniform dispersion of PLGA within the solvent or polymer solution. By ensuring the successful emulsification of the components, the subsequent steps of the fabrication process can proceed smoothly [51]. Following emulsification, the emulsified ethyl acetate (or other organic solution) droplets are then hardened. These hardened droplets serve as pores or void spaces in the co-solvent/extraction polymer. The formation of these porous structures is significant as it impacts the overall characteristics of the resulting hydrogel. The porosity and void space allow for the effective loading and release of drugs or other bioactive agents within the hydrogel matrix. Importantly, the one-step particle-leaching process employed in this technique is suitable for a broad range of polymers [52]. 

This versatility makes it highly applicable in various biomedical and tissue engineering applications. Additionally, the particle-leaching process is scalable, allowing for the fabrication of hydrogels with a general microsphere size. This scalability is advantageous as it permits the production of hydrogels in different sizes, catering to specific requirements and applications. The emulsion method is the most widely used method for preparing PLGA hydrogels and provides a versatile platform for biomedical applications [53]. By immersing a crosslinked porous polymer into PLGA dissolved in a suitable non-toxic solvent, and subsequently evaporating the solvent while initiating the crosslinking process, a single-phase hydrogel with open channels can be obtained. Researchers have reported various strategies to produce desirable PLGA hydrogels using the emulsion method, often involving the incorporation of surfactant dispersion. This allows the formation of stable emulsion droplets which eventually harden into the desired heterogeneous solid matrix, offering enhanced mechanical properties and controlled release capabilities [54].

### 2.5. Solvent Casting

Generally, a PLGA matrix is formed by solvent evaporation or diffusion. The most commonly used method, solvent casting, has been widely employed to mold various forms of PLGA matrices, including films, foils, fibers, and microparticles. In the solvent casting process, the polymer, along with other dissolved bioactive agents, is dissolved in commonly used solvents, such as chloroform and acetone, to obtain a homogeneous solution. This solution is then cast into a suitable mold or spread onto a substrate. As the solvent gradually migrates from the polymer/solvent solution to the surrounding air, a solid film is formed. During the solvent evaporation process, the solvent present in the polymer solution diffuses towards the air/polymer interface, resulting in the formation of a flat film [41]. It is worth noting that by manipulating the surface properties, the cross-section of the films can be significantly altered. This can lead to the formation of distinct features, such as finger-like layers or porous microsponges within the PLGA matrix [52]. Through careful optimization, an ideal release profile for hydrophobic drugs encapsulated within the solvent casting systems has been achieved. However, it is important to address the challenges associated with organic dissolution or phase separation, as they can hinder the sustained release of bioactive agents from the PLGA matrix. The major drawback is that the hydration and degradation of solvent casting systems are often insufficient to provide effective release characteristics [55]. This limitation significantly hinders the potential applications of these systems. Consequently, many researchers around the world have tirelessly explored and experimented with various approaches to overcome this deficiency. Unfortunately, despite their efforts, the release characteristics of the resulting systems has still turned out to be flawed in several respects. In response to this challenge, there have been promising advancements in the field of medicated polymer matrices and other modified systems. These advances aim to improve the delivery characteristics of therapeutic administrations by incorporating innovative concepts and technologies. However, it is important to note that these potential strategies are currently limited by an incomplete understanding of the fundamental mass transport and mechanistic interactions of the polymer matrix [56]. To address these limitations and “unlock” the full potential of therapeutic administrations, various strategies have been explored. For instance, after the films are dried, the hydrophobic polymer and any residual virtual solvent are allowed to evaporate, resulting in the formation of a PLGA nano-fiber matrix. This nano-fiber matrix has shown promising results in effectively encapsulating small water-insoluble drugs within its rough surface morphology. However, it should be noted that the sustained release of drugs from this matrix is still an area that requires further investigation. In order to achieve sustained or semi-sustained release, as well as zero-order release kinetics, additional hydrophobic additives can be blended with the polymer/solution and encapsulated drugs [31]. The incorporation of these additives allows for variations in the polymer chains or the crystallinity of the polymer matrix, thereby enabling control over the drug release. This approach has shown potential in expanding the capabilities of PLGA matrices and tailoring their release profiles for different therapeutic applications [56].

### 2.6. Electrospinning 

Using electrospinning to obtain PLGA hydrogels has emerged as a versatile and promising technique for achieving sustained drug delivery in biomedical applications. This method combines the inherent properties of PLGA polymers with the unique advantages of electrospinning, enabling the production of nano- to micro-scale fibers with controlled drug release profiles. Electrospinning uses an electric field to draw or melt a polymer solution into ultrafine fibers. The process typically involves a syringe or pump to deliver the polymer solution through a needle or spinneret, which is connected to a high-voltage power supply. When the electric field is applied, electrostatic forces overcome the surface tension of the polymer solution, forming a jet that expands and solidifies into fibers as the solvent evaporates or coagulates in a collection medium. The resulting fibers can range in diameter from nanometers to micrometers and offer a high surface area-to-volume ratio and a porous structure that promotes drug loading and release [57].

The advantages of electrospinning include controlled release profiles, high drug loading capacity, biocompatibility, biodegradability, ease of scalability, and versatility [58]. Electrospun PLGA fibers allow for precise control over the release kinetics of encapsulated drugs. Factors such as polymer composition, fiber morphology, and drug loading can be tailored to achieve sustained release over extended periods. The high surface area and porous structure of electrospun fibers facilitate the efficient encapsulation of a wide range of drugs, including hydrophobic and hydrophilic compounds, proteins, and growth factors [59]. Electrospinning can incorporate multiple drugs or bioactive agents within the same scaffold, enabling combination therapies or the sequential release of different agents. Moreover, it is a scalable technique that can be adapted for the industrial production of drug delivery systems, ensuring reproducibility and uniformity in fiber morphology and drug release characteristics [60].

Despite its advantages, electrospinning presents some challenges, such as process optimization, potential drug degradation, or poor mechanical properties. Parameters such as polymer concentration, solvent selection, voltage, and the distance between spinneret and collector must be carefully optimized to achieve the desired fiber morphology and drug release kinetics [61]. Some drugs may degrade or lose activity during the electrospinning process due to exposure to solvents, electric fields, or prolonged processing times. Electrospun fibers may exhibit lower mechanical strength compared to bulk materials, requiring strategies to enhance scaffold stability and durability [60]. 

Electrospun PLGA hydrogels have been utilized in various biomedical applications, including wound healing [62], for the delivery of growth factors and antimicrobial agents to promote tissue regeneration and prevent infection [58]. In orthopedics, electrospun PLGA hydrogels have been utilized to control the release of analgesics and anti-inflammatory drugs for the management of musculoskeletal disorders such as osteoarthritis [43]. In neuroscience, electrospun PLGA hydrogels have been applied for the localized delivery of neuroprotective agents or growth factors for the treatment of neurodegenerative diseases or spinal cord injuries [63]. The electrospinning of PLGA hydrogels represents a promising strategy for achieving sustained drug delivery in biomedical applications. By harnessing the advantages of PLGA polymers and electrospinning technology, researchers can tailor drug release profiles to meet specific therapeutic needs while ensuring biocompatibility and biodegradability. Continuous advances in materials science and process optimization are expected to further improve the efficacy and clinical implementation of electrospun PLGA hydrogel-based drug delivery systems.

### 2.7. Thermal Gelation

Thermal gelation of PLGA hydrogels is a versatile technique that leverages the thermoresponsive properties of PLGA to form hydrogels under controlled temperature conditions. PLGA hydrogels are typically prepared using block copolymers or blends where PLGA serves as the hydrophobic segment. Below a critical gelation temperature, these polymers remain in solution due to their solvency in aqueous environments. However, upon reaching the gelation temperature, which is often slightly above physiological temperatures (~37 °C), the polymer undergoes a phase transition from a liquid solution to a gel state. This transition occurs due to the dehydration of the polymer chains and subsequent physical entanglement or aggregation, resulting in the formation of a three-dimensional structure [48].

One of the significant advantages of thermal gelation is its injectability. The polymer solution can be injected as a liquid precursor into the target site within the body, where it undergoes gelation upon exposure to body heat. This property makes it suitable for minimally invasive procedures [64]. The gelation process itself is non-toxic and does not require harsh chemicals or crosslinking agents that could potentially harm biological tissues [65]. The gel structure formed by thermal gelation can encapsulate drugs or bioactive molecules. The sustained release of these substances can be achieved by modulating the polymer composition and gelation parameters, ensuring controlled drug delivery over a prolonged period of time [65].

The thermal gelation of PLGA hydrogels has been employed in various biomedical fields, including controlled drug delivery systems, tissue engineering scaffolds, and wound healing applications. Its adaptability allows for the incorporation of different therapeutic agents tailored to specific medical needs [66].

However, there are some challenges that should be taken into account. In recent years, researchers have focused on improving the delivery systems for these materials, particularly in bone regenerative medicine. Although hydrogels have shown promise in this regard, their application has been limited to areas with small cavities and irregular shapes [67]. This is mainly due to the challenges associated with their administration and distribution within the targeted region. Interstitial injections of hydrogels have proven to be a complex and intricate procedure. Precise delivery of these materials to the intended location requires surgical expertise and meticulous care. Furthermore, both therapeutic agents and PLGA materials require high drug loads, zero-order release, easier handling during treatment, and careful design and physical forms for efficient drug delivery. To meet these needs, scientists have developed thermoresponsive hydrogel PLGA for cellular or therapeutic drug delivery which belongs to a novel class of biological gels with broad applications in tissue regeneration, drug delivery, and numerous other areas of biomedicine. In general, thermoresponsive PLGA gels exhibit liquid properties at both low and high temperatures. However, when exposed to human body temperature, the solution quickly turns into a non-finishing semi-liquid gel, improving its performance as a drug delivery system [68]. 

Furthermore, when compared to the use of simple PLA/PLGA microspheres, hydrogels have shown certain drawbacks. These include a lower drug load capacity, a peak release rate, and potential irradiation of the therapeutic agent. Such shortcomings can significantly impact the chemical and physical properties of the drug, jeopardizing its efficacy and overall therapeutic outcome. Considering these observations, ongoing research aims to overcome these limitations and optimize the use of PLA and PLGA for biomedical applications. Scientists are exploring new approaches to maximize drug loading, regulate release rates, and minimize adverse effects on the therapeutic agent. By overcoming these challenges and refining the chemical and physical properties of PLA and PLGA, researchers hope to unlock their full potential in various therapeutic applications [68].

The gelation temperature must be precisely controlled to ensure gel formation occurs at the intended physiological conditions. Variations in temperature or polymer concentration can affect the gelation kinetics and mechanical properties of the hydrogel. While PLGA hydrogels formed by thermal gelation are injectable, they may have lower mechanical strength compared to crosslinked hydrogels. Strategies to improve mechanical properties without compromising injectability are being actively researched. The degradation rate of PLGA hydrogels must be compatible with the intended application. The balance between degradation kinetics and drug release profiles is crucial for maintaining therapeutic efficacy over the desired timeframe [67].

The thermal gelation of PLGA hydrogels is used to encapsulate and deliver drugs for localized therapies, such as cancer treatment [69] or chronic wounds [70]. These hydrogels serve as scaffolds to support cell growth and tissue regeneration, particularly in musculoskeletal and cardiovascular applications [71]. In ocular applications, thermal gelling PLGA hydrogels have been investigated for sustained drug release to treat diseases such as glaucoma or macular degeneration [72]. Continued advances in materials science and gelation techniques are expected to further increase the utility and effectiveness of these hydrogels in diverse therapeutic applications [67]. 

### 2.8. Photopolymerization 

Photopolymerization of PLGA hydrogels has emerged as a sophisticated approach to precisely control drug release kinetics and spatial distribution in biomedical applications. Photopolymerization involves the use of light-sensitive molecules (photoinitiators) to initiate/trigger polymerization reactions in the presence of monomers or pre-polymers. For PLGA hydrogels, photopolymerization typically involves the functionalization of PLGA with photoreactive groups, such as methacrylate or acrylate moieties, that can crosslink upon exposure to light of specific wavelengths (typically ultraviolet or visible light). The photoinitiator absorbs photons from light and generates reactive species (free radicals or ions) that trigger polymerization and crosslinking reactions, forming a three-dimensional hydrogel network. This process occurs quickly and can be spatially and temporally controlled, enabling the precise encapsulation and release of drugs [73].

The advantages of photopolymerization for PLGA hydrogels involve spatial and temporal control, high efficiency and versatility, tunable properties, and integration with biomolecules for biocompatibility and biodegradability [74].

Photopolymerization enables the localized crosslinking of PLGA hydrogels within seconds to minutes upon exposure to light, allowing for precise control over the spatial distribution of drug-loaded hydrogel matrices. The reaction occurs quickly and efficiently under mild conditions, preserving the bioactivity of sensitive drugs or biological molecules encapsulated within the hydrogel matrix. The mechanical and degradation properties of photopolymerized PLGA hydrogels can be tailored by adjusting parameters such as polymer concentration, photoinitiator type and concentration, light exposure intensity, and duration. PLGA is biocompatible and biodegradable, making photopolymerized PLGA hydrogels suitable for sustained drug delivery applications without inducing significant inflammatory responses [75]. 

Photopolymerization allows for the incorporation of bioactive molecules, such as growth factors or peptides, into the hydrogel matrix, facilitating synergistic therapeutic effects or tissue regeneration. Despite its advantages, the photopolymerization of PLGA hydrogels presents several challenges: depth of light penetration, photoinitiator toxicity, and shelf stability. Limited light penetration depth restricts the thickness of photopolymerized hydrogels, necessitating strategies for creating thicker constructs for certain applications. Some photoinitiators can be cytotoxic or cause photodamage to encapsulated drugs or biomolecules, requiring careful selection and optimization. Photopolymerizable formulations may require careful handling and storage to maintain photoinitiator stability and prevent premature polymerization [73].

Photopolymerized PLGA hydrogels have been applied in various biomedical fields, including regenerative medicine, for the controlled release of growth factors or stem cell therapies for tissue engineering [76,77] and wound healing applications [78]. In drug delivery systems, photopolymerized PLGA hydrogels have been used for the sustained release of antibiotics, anti-inflammatory drugs, or chemotherapeutic agents for localized treatment of infections, inflammation, or cancers [79,80]. 

The photopolymerization of PLGA hydrogels represents a powerful tool for developing advanced drug delivery systems with precise spatial and temporal control of drug release. By leveraging the unique properties of PLGA polymers and photopolymerization technology, researchers can develop innovative therapies that have greater efficacy, reduced side effects, and improved patient outcomes in various clinical settings [75].

## 3. Properties of PLGA Hydrogels

**Scheme 1:**
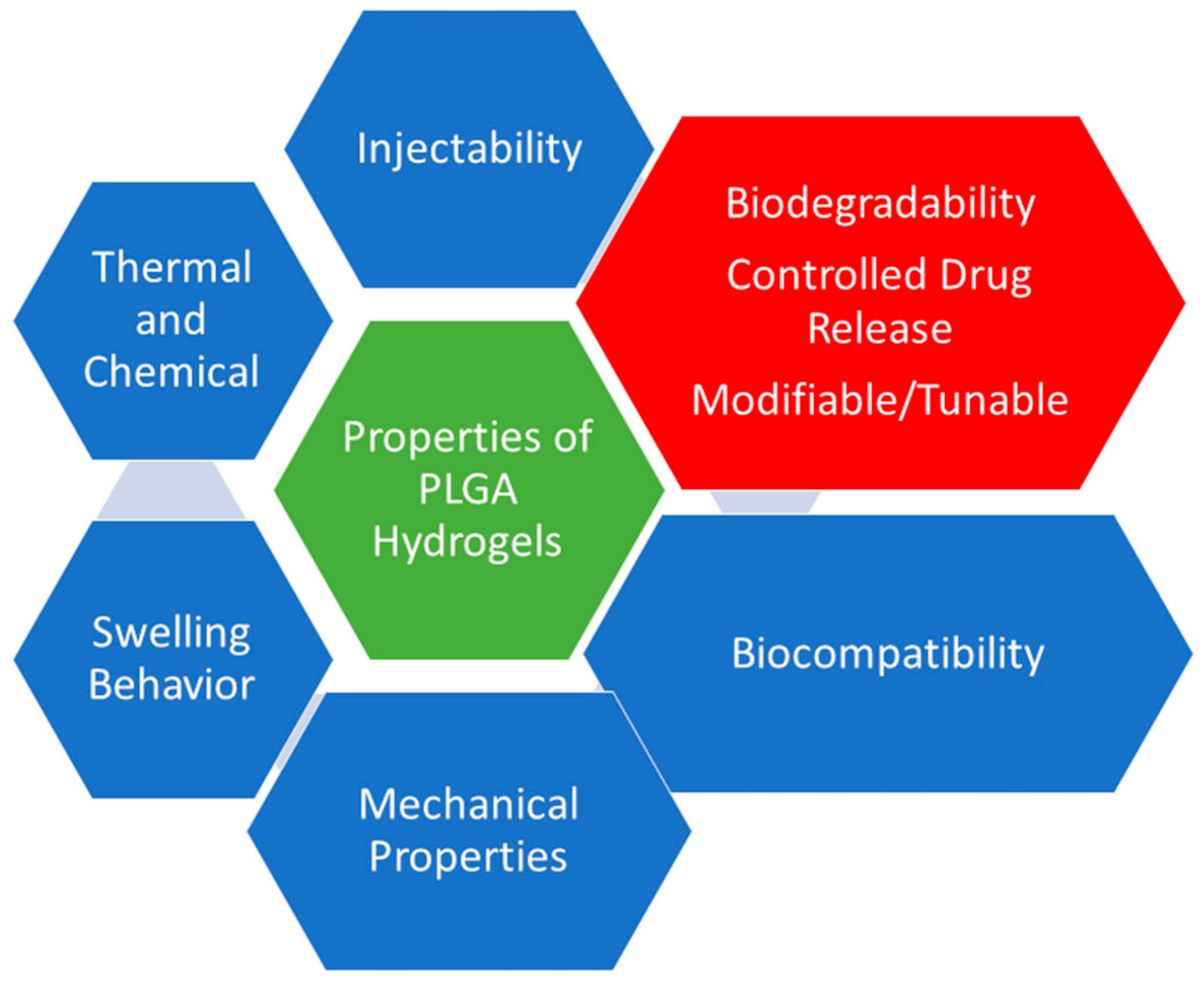
Properties of PLGA hydrogels.

PLGA hydrogels (Scheme 1) exhibit several beneficial properties that make them suitable for various biomedical applications, particularly in drug delivery and tissue engineering. Below are the key properties of PLGA hydrogels, each accompanied by example cases where PLGA hydrogels have been used for sustained drug release.

### 3.1. Biodegradability

PLGA hydrogels break down into lactic acid and glycolic acid, which are naturally metabolized by the body. The rate of degradation can be controlled by adjusting the ratio of lactic acid to glycolic acid in the copolymer [81]. PLGA hydrogels have been used to create an injectable depot system for the sustained release of insulin. This system provides a steady release of insulin over an extended period, reducing the frequency of injections that diabetics need [31].

In another example, PLGA hydrogels loaded with bone morphogenetic proteins (BMPs) have been applied to sustain the release of these proteins, promoting bone regeneration in orthopedic applications [82].

### 3.2. Biocompatibility

PLGA hydrogels are highly biocompatible, meaning they do not induce significant inflammatory or immune responses when implanted in the body [83].

PLGA hydrogels have been used to deliver chemotherapeutic drugs like paclitaxel in a sustained manner directly to the tumor site, reducing systemic toxicity and improving therapeutic outcomes [53,84].

For the treatment of ocular diseases, PLGA hydrogels have been used to ensure the sustained release of anti-inflammatory drugs and improve patient compliance by reducing the need for frequent eye drops [85,86].

### 3.3. Mechanical Properties

Variating the polymer composition, crosslinking density, and the addition of other materials can tailor the mechanical properties of PLGA hydrogels.

PLGA hydrogels designed with appropriate mechanical properties have been used to deliver growth factors for cartilage repair, providing a scaffold that supports the mechanical loads while promoting tissue regeneration [87]. 

In wound healing, PLGA hydrogels with adjustable mechanical properties have been used to sustain the release of antimicrobial agents, forming a protective barrier while promoting healing [88].

### 3.4. Controlled Drug Release

PLGA hydrogels provide controlled and sustained drug release which can be fine-tuned by manipulating the degradation rate of the PLGA, the hydrogel’s porosity, and the swelling behavior [89].

PLGA hydrogels loaded with antibiotics such as vancomycin have been used to treat bone infections, to ensure sustained release of the drug at the infection site, and to improve the effectiveness of the treatment [90].

For postoperative pain management, PLGA hydrogels have been employed to deliver analgesics like bupivacaine over a prolonged period, reducing the need for frequent dosing [91].

### 3.5. Swelling Behavior

PLGA hydrogels swell upon contact with aqueous environments, which is important for drug loading and release, as well as providing a hydrated environment for cell growth in tissue engineering applications [92].

PLGA hydrogels with tailored swelling properties have been used for the sustained release of hydration-sensitive drugs, ensuring that the drug release profile is controlled by the swelling behavior of the hydrogel [93].

In tissue engineering, PLGA hydrogels have been utilized to encapsulate cells and provide a sustained release of growth factors, thereby improving cell viability and proliferation [94].

### 3.6. Thermal and Chemical Stability

PLGA hydrogels are generally stable under physiological conditions, maintaining their structure and function at body temperature and allowing for sterilization using standard methods without significant degradation [95].

PLGA hydrogels stable at body temperature have been used to deliver osteogenic factors for bone tissue engineering, maintaining the release kinetics over several weeks [96,97].

PLGA hydrogels that can be sterilized without degradation have been developed for the sustained delivery of anti-inflammatory drugs in arthritic joints [98].

### 3.7. Modifiable/Tunable Surface Properties

The surface properties of PLGA hydrogels can be modified to enhance cell attachment, proliferation, and differentiation, making them ideal for creating scaffolds in tissue engineering [99].

PLGA hydrogels functionalized with RGD peptides have been used to improve cell adhesion and promote sustained release of growth factors in wound healing applications [100,101].

PLGA hydrogels coated with nanoparticles have been employed for the sustained release of anti-cancer drugs, improving the targeting and efficacy of the treatment [102].

### 3.8. Tunable Degradation Kinetics

Adjusting the copolymer composition and molecular weight can fine-tune the degradation kinetics of PLGA hydrogels, allowing for the design of hydrogels that degrade over a specific period [103].

PLGA hydrogels with tailored degradation rates are used for the sustained release of hormones such as leuprolide acetate in hormone replacement therapy, ensuring consistent hormone levels for months [104,105].

In vaccine delivery, PLGA hydrogels have been designed to degrade slowly, providing a sustained release of antigens and enhancing the immune response [106,107].

### 3.9. Injectability

PLGA hydrogels can be formulated as injectable systems, providing a minimally invasive method for delivering drugs or cells to specific sites in the body [108].

Injectable PLGA hydrogels have been developed for the sustained release of local anesthetics, providing long-lasting pain relief after surgery [25,109].

PLGA hydrogels formulated for injection have been used to deliver stem cells to damaged tissues, where the hydrogel provides a sustained release of growth factors to support cell survival and differentiation [110,111].

## 4. Sustained Release Mechanisms of PLGA Hydrogels

PLGA hydrogels enable controlled drug release through various kinetic mechanisms influenced by factors such as pH, monomer ratio, and intrinsic viscosity of PLGA. Understanding these factors is crucial for optimizing drug delivery systems to achieve the desired therapeutic outcomes.

**Figure 5:**
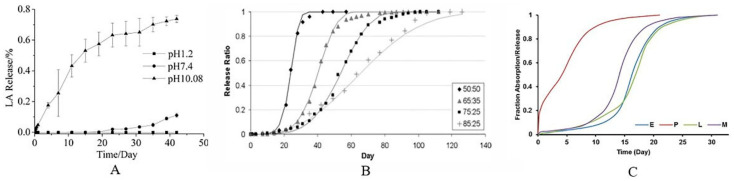
(**A**) Comparison of PLGA degradation rates under different pH conditions. (**B**) Comparison of PLGA degradation rates for different monomer ratios. (**C**) Comparison of the degradation rates of PLGAs with different intrinsic viscosities; the magnitude of the intrinsic viscosities of the four PLGAs is P < L < E < M [112].

The degradation rate of PLGA is significantly influenced by the pH of the environment (Figure 5). As demonstrated by Lu et al. [112], PLGA degrades faster under acidic conditions compared to neutral or basic conditions. This accelerated degradation is due to the hydrolysis of ester bonds in PLGA, which occurs more rapidly in acidic environments. Thus, the pH of the medium is a critical factor in designing PLGA-based drug delivery systems for specific applications [113].

The ratio of lactic acid to glycolic acid in PLGA also impacts its degradation rate. Lu et al. [112] illustrate that PLGA with a higher glycolic acid content degrades more quickly than those with higher lactic acid content. This is because glycolic acid contributes to increased hydrophilicity and amorphous characteristics, enhancing water uptake and hydrolysis rates. Adjusting the monomer ratio allows for the customization of degradation and drug release rates according to therapeutic needs [114].

Intrinsic viscosity, indicative of the molecular weight of PLGA, affects its degradation behavior. It was demonstrated that PLGAs with different intrinsic viscosities degrade at varying rates, with the order being P < L < E < M. Higher intrinsic viscosity correlates with a slower degradation rate due to the increased molecular weight, resulting in more extended drug release profiles [115].

The drug release kinetics from PLGA hydrogels can be modulated by manipulating the factors mentioned above. The release mechanisms typically include diffusion, degradation, or a combination of both.

Initially, drug release is often governed by diffusion through the PLGA matrix. This phase may exhibit a burst release, where a significant portion of the drug is rapidly released from the hydrogel’s surface.

As the PLGA matrix degrades, the release mechanism transitions to being degradation-controlled. Here, the drug release rate aligns with the degradation rate of the PLGA, which can be fine-tuned by altering the pH, monomer ratio, or intrinsic viscosity.

In many practical applications, the drug release profile is a combination of diffusion and degradation mechanisms. Early stages might be diffusion-dominated, with degradation playing a more significant role as the matrix breaks down over time.

**Figure 6:**
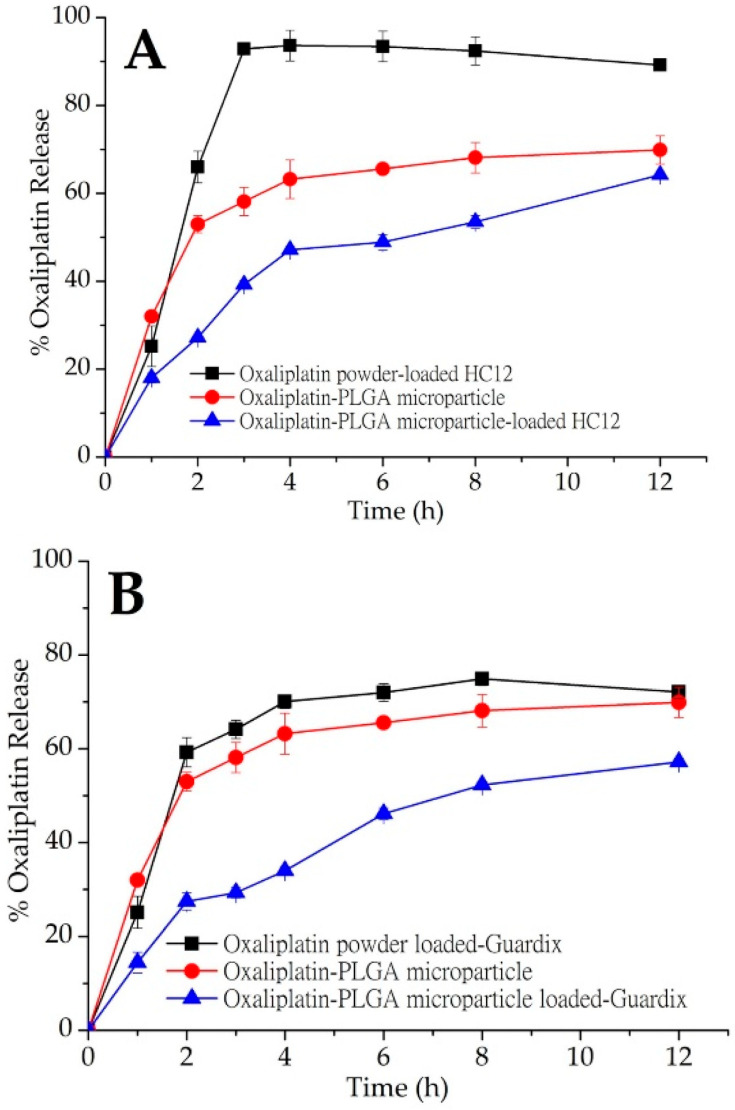

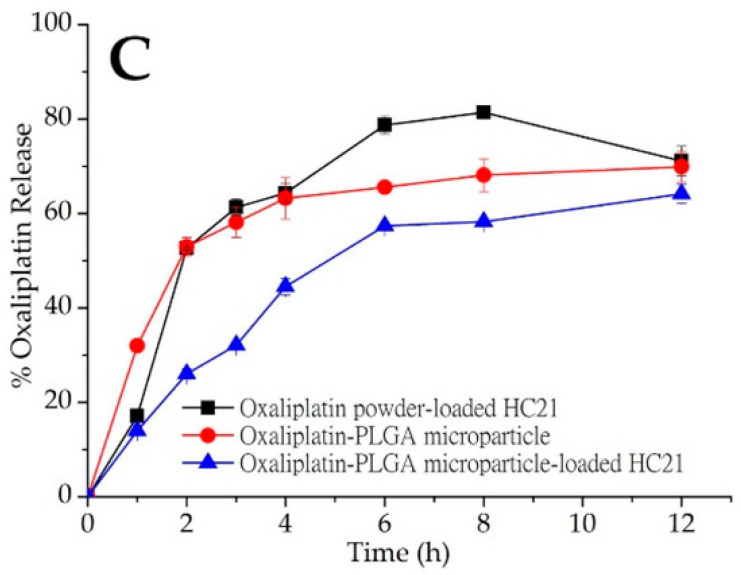
In vitro oxaliplatin release from PLGA microparticles (red, ●). Oxaliplatin release profile from oxaliplatin powder and oxaliplatin–PLGA microparticles-loaded in (**A**) HC12; (**B**) Guardix-Sol^®^; and (**C**) HC21 hydrogel. Mean ± S.D. (n = 3) [116].

The practical application of these concepts can be seen in the release profile of oxaliplatin from PLGA microparticles. As shown in Figure 6, oxaliplatin release from PLGA microparticles exhibits a sustained release over time compared to oxaliplatin powder. When these microparticles are loaded into different hydrogels, such as HC12, Guardix-Sol^®^, and HC21, the release profiles differ significantly (A, B, and C, respectively), highlighting the impact of the hydrogel matrix on drug release kinetics. These findings underscore the potential of PLGA-based systems in achieving controlled and sustained drug delivery [116].

Understanding the mechanisms governing the release of therapeutic agents from PLGA hydrogels is crucial for optimizing their performance in biomedical applications. The release mechanisms, including diffusion, degradation, and swelling, can be manipulated through the careful selection of polymer properties and hydrogel formulation. Advances in understanding these mechanisms continue to drive innovations in drug delivery systems, paving the way for more effective and personalized therapies.

### 4.1. Diffusion-Controlled Drug Release System

One of the main mechanisms of drug release from PLGA hydrogels is diffusion. In this process, molecules are released as a result of their movement through the aqueous pores of the hydrogel matrix. In the initial phase, a burst release takes place. When a drug-loaded PLGA hydrogel is exposed to the physiological environment, an initial burst release occurs. This phase is characterized by a rapid release of a significant amount of drug molecules that are loosely bound or located near the surface of the hydrogel. The mechanism implies that drug molecules diffuse through the aqueous channels and pores within the hydrogel matrix. The release rate is initially high due to the steep concentration gradient between the drug-loaded hydrogel and the surrounding medium.

In a later phase, a sustained release occurs. As diffusion continues, the release rate gradually decreases. This sustained release phase is governed by Fick’s law of diffusion, where the rate of release depends on the concentration gradient, the drug’s diffusion coefficient, and the thickness of the hydrogel matrix [117].

The diffusion rate can be influenced by the polymer composition (e.g., the ratio of lactic acid to glycolic acid in the PLGA), the molecular weight of the polymer, and the physicochemical properties of the drug (e.g., size and hydrophilicity). The diffusion rate depends on factors such as the molecular size, hydrophilicity, and mesh size of the hydrogel network. Smaller molecules typically diffuse faster than compared to larger ones, resulting in faster release rates over time [31]. The natural or artificial barriers, such as polymer matrices encapsulating drugs or transplanted devices, interact with the drug release scaffold from both the interior and exterior directions. For the drug delivery system of interest, the two flow directions can result in two different control mechanisms: inside-out could induce diffusion-controlled drug release, while the reverse flow from outside-in would cause hydration-controlled or erosion-controlled drug release. When the diffusional release of the drug is controlled, the drug molecules are transported through the polymer matrix, which serves as a rate-limiting barrier, thus forming a diffusion barrier with different properties and configurations [1].

Among various possible mechanisms affecting the release of drugs incorporated into controlled delivery systems, the diffusion-controlled release is one of the most important and effective. The diffusion of drug molecules from the polymer matrix into the surrounding aqueous environment and into the receiving phase or tissues that act as drug sinks could sustain needed release profiles, particularly in the context of pulsatile, continuous, or on-demand delivery [118].

#### 4.1.1. Degradation-Controlled Release System

Degradation-controlled release is another mechanism that often contributes to a prolonged and sustained release of drugs as the hydrogel continues to degrade and erode. PLGA hydrogels degrade through the hydrolysis of ester bonds in the polymer backbone, leading to a gradual breakdown of the matrix [117]. When the polymer degrades, it releases encapsulated drugs, proteins, or small molecules. This degradation-controlled release mechanism can be tuned by adjusting the composition of the PLGA (e.g., the lactide to glycolide ratio), molecular weight, and the degree of crosslinking. A higher crosslinking density generally slows down the degradation and release rate, offering sustained release profiles. By the completion of the delivery through surface erosion that enables the newly formed surface at the interface with the body fluid to disestablish as a potential barrier to mass transfer, the erosion mechanism can be used to foretell when the delivery system transfers to a zero-order drug release regime. Additionally, in certain cases of passive diffusion of biomolecules, erosion specifically occurs in the tumor due to localized factors such as increased enzyme activity, acidic pH, and elevated metabolic rates. These conditions accelerate the degradation of the hydrogel matrix in the tumor environment, thereby promoting localized erosion and targeted drug release. Therefore, degradation-controlled release is a potential mechanism to contribute to anti-cancer activity [119].

One of the most popular mechanisms for rate control is to prevent drug leakage through physical or chemical encapsulation, such as covalent bonding between drugs and PLGA hydrogels. This modulation of the drug release kinetics can be based mainly on drug release during the biodegradation of the hydrogels and is referred to as “degradation-controlled release”. The degradation rate of the hydrogel mainly depends on the species and structure of the material. The PLGA provides -(CH_2_-CO-O)- units within the framework for the rapid degradation into CO_2_ and H_2_O molecules, finally leading to a decrease of the delivery system by “bulk erosion” [31].

#### 4.1.2. Swelling-Controlled Release System

Swelling-controlled release of PLGA hydrogels in aqueous environments can also influence the kinetics of drug release. Upon contact with physiological fluids, hydrogels absorb water and swell, increasing the mesh size and facilitating the diffusion of encapsulated molecules. The swelling behavior of PLGA hydrogels depends on parameters such as polymer concentration, pH, and temperature, all of which can be individually adjusted to achieve specific release profiles [31].

#### 4.1.3. Combined Release Mechanisms

Combined mechanisms can occur. In many cases, drug release from PLGA hydrogels involves a combination of diffusion and degradation mechanisms. During the burst release phase, diffusion initially dominates. As degradation progresses, the erosion of the hydrogel matrix becomes more significant and affects the release kinetics over time [41]. Often, drug release from PLGA hydrogels also involves a combination of diffusion, degradation, and swelling mechanisms. For instance, initial burst release may occur due to rapid diffusion of molecules near the surface, followed by sustained release as degradation progresses and the hydrogel swells. Understanding and optimizing these mechanisms are crucial for the development of PLGA hydrogels that meet the desired therapeutic needs, such as controlled release over extended periods or targeted delivery to specific tissues.

### 4.2. Types of Kinetics of Drug Release Mechanisms

The kinetics of drug release from PLGA hydrogels are pivotal in development of controlled drug delivery systems. In general, hydrogels are porous and possess interconnected pore networks, and drug release occurs through a mixture of molecular diffusion, convective flow, polymer swelling, and relaxation behavior. Therefore, the main drug transport mechanism in most practical hydrogel drug delivery systems is either Fickian diffusion, non-Fickian diffusion, case-II transports, or a combination of two in the same hydrogel system. In this context, clarifying the kinetic types is important for a better understanding and efficient application of PLGA hydrogels in drug delivery. Understanding these mechanisms allows for the design of hydrogels that can provide sustained and targeted release of therapeutic agents. Next, we shortly examine four key models: zero-order kinetics, first-order kinetics, the Higuchi model, and the Peppas model.

#### 4.2.1. Zero-Order Kinetics

Zero-order kinetics is characterized by a drug release rate that remains constant over time, regardless of the drug concentration in the hydrogel. This type of release mechanism is highly sought after in therapeutic applications because it can maintain constant drug levels in the bloodstream and minimize the peaks and troughs that can occur with other release mechanisms [120]. Zero-order drug release is independent of the initial drug concentration or the dimensions of the hydrogel configuration, such as the disc or wafer tested. It is the optimal release kinetics because the kinetics model and parameters for drug release are predictable, attainable, and applicable to controlled drug delivery. Dose-independent, and decoupled from any initial burst release of the drug. The release criteria for zero-order are that n < 0.43 for erosion and r^2^ > 0.98 for drug release. When the release profile is zero-order, the release criteria of zero-order kinetic release can only be obtained with PLGA containing 50% carbopol which is 20% esterified. With the zero-order release kinetic profile, the required mass transport of the drug is uniform, reaching the release area. The drug release is decoupled from the hydrogel matrix gel erosion [121].

Since the release rate is independent of the rate of degradation of the non-drug-filled matrix degradation, zero-order kinetics is generally considered to be the preferred kinetic release profile for a sustained release system. The drug release from the hydrogel is controlled by Fickian diffusion. When the polymer degradation and drug release are decoupled, the in vitro drug release can be rapid, reaching that of the erosion by area. The release criterion is r^2^ > 0.98 with n approaching 1. The release units for zero-order kinetics are μg/(mm^2^day). The zero-order release rate and period are independent of the initial amount of drug in the hydrogel system or the conditions of the experiment, making it the most predictable release kinetics [122].

The selection of the polymer composition is crucial. Variations in the ratio of lactic acid to glycolic acid in PLGA can influence the degradation rate, which is critical for achieving a zero-order release profile [123]. The geometric shape and size of the hydrogel matrix can influence the surface area available for drug release. For instance, cylindrical or disk-shaped matrices could provide more uniform, consistent release rates [124].

A higher initial drug loading can support a zero-order sustained release because the larger drug reservoir helps maintain the concentration gradient necessary for constant release. The extent of crosslinking within the hydrogel affects its degradation rate and mechanical properties, thereby influencing the release rate. Higher crosslinking can slow down degradation, supporting a more consistent release rate [125].

The excipients added, such as polyvinyl pyrrolidone (PVP), hydroxypropyl-β-cyclodextrin (HP-β-CD), polyethylene glycol (PEG), and Span 80, have a noticeable effect on the drug release kinetics [1].

#### 4.2.2. First-Order Kinetics

First-order kinetics describes a drug release mechanism in which the release rate is directly proportional to the drug concentration remaining in the hydrogel. This results in an exponential decrease in the release rate over time, which is typical of many conventional drug delivery systems [126].

Drugs with higher solubility within the hydrogel matrix are more likely to follow first-order kinetics because they can easily diffuse out of the matrix [127].

The degradation rate of the PLGA polymer influences the rate at which the drug is released. Faster degradation can lead to a quicker release [128].

According to the principles of first-order kinetics, higher initial drug concentrations will result in higher initial release rates. The drug’s diffusion coefficient within the hydrogel matrix is a critical factor that affects the release kinetics. Higher diffusion coefficients generally result in faster release rates [129].

As long as the kinetic of the phenomenon is slow or only the release of the drug through the external environment is not a thermodynamic process, the equilibrium is never reached, and the spontaneous mixing process cannot take place through the hydrogel network. From the kinetics of drug release, much information is derived on both native hydrogel and the complex drug/hydrogel. The methods of studying the release kinetics from hydrogel depend on many factors, including the nature of the hydrogel, the hydrogel network size, the diffusion of the released molecules, hydrophobic/hydrophilic interactions, electrostatics, steric spheres, and a chain relaxation of hydrogel. The release time depends greatly on the degree of drug loading, the approach to drug equilibrium, and the ability loading to the drug. An understanding of the release mechanism is necessary in the choice of polymers that minimize the initial burst release of a loaded pharmacological agent [130].

Kinetics describes the speed, the order, and the reaction mechanisms with time of the reaction. Kinetic studies allow not only the understanding of the nature of the drug–hydrogel interactions but also the prediction of the hydrogel model needed for different pharmaceutical applications [131]. The evaluation of the kinetics of drug release from the hydrogel is useful to understand the physical–chemical mechanisms involved in the transport of drugs (and other substances) from the environmental bulk, through the hydrogel network, and its further release to the external environment [132].

The diffusion and degradation processes both follow first-order kinetics, and PLGA hydrogels display zero-order kinetics if there is no erosion. The hydrogel displays a prolonged release effect. Its release curve can be divided into the first-order and zero-order release segments, which are influenced by multiple factors [133].

### 4.3. Higuchi Model

The Higuchi model is the most widely used mass transport equation for drug release in polymer-based drug delivery matrix systems. The Higuchi model for drug release from polymeric solid devices is actually a special case of the diffusion equation and assumes only one-dimensional regular drug distribution in the polymer matrix. It is actually a limiting case of a form of the diffusion equation with fixed concentration boundary conditions at the two infinite parallel surfaces of the slab. Considering this, the Higuchi model is often considered as a square root of time-driven diffusion. Assuming no polymer relaxation, trans-polymer drug release, dose metrics, and cylindrical geometry (radius < thickness), Higuchi’s first-dimensional application of Fick’s second law of diffusion [134]. Specifically, the Higuchi model describes the release of active ingredients from a matrix system, where the release rate is proportional to the square root of time. This model assumes a homogeneous matrix and is often used for systems where diffusion is the primary release mechanism [128].

The model is applicable to drugs with limited solubility within the matrix. Highly soluble drugs might deviate from the model due to too rapid a release [135]. The initial porosity and the changes in porosity due to matrix erosion can influence drug release rates. A more porous matrix facilitates quicker drug diffusion [136]. 

The Higuchi model assumes a uniform drug distribution within the matrix. A non-uniform distribution can lead to deviations from the expected release profile.

The drug’s diffusion coefficient within the polymer matrix is a critical parameter. The polymer’s physical and chemical properties can influence this [137].

### 4.4. Peppas Model

The Peppas model, also known as the Korsmeyer–Peppas model, is an empirical model used to describe drug release from polymeric systems when the mechanism of release is not well-known or involves multiple processes [137].

The model is described by Equation (1):

M_t_/M_∞_ = kt^n^, (2)

where M_t_/M_∞_ is the fraction of drug released at the time, t, k is a release rate constant, and n is the release exponent indicative of the mechanism of release [127].

The degree of swelling can significantly influence the release mechanism, particularly in the transition from diffusive to swelling-controlled release [127].

Non-uniform distribution of the drug within the matrix can cause deviations from the expected release profile [127].

The rate at which the polymer degrades impacts the release exponent, *n*. Faster degradation can lead to a higher release rate [127].

The density of crosslinks within the hydrogel matrix affects the drug diffusion rate and polymer erosion, which are critical for the Peppas model [137]. The dissolution and release mechanism of the drug from the polymer matrix always involves the mass transfer process of the solvent into the polymer and the redistribution of the drug in the matrix, forming a two-phase system containing drug-rich and polymer-rich phases. Generally, the Peppas model is used to analyze solute mobility, an empirical model that is very efficient for investigating and predicting the efficiency of polymers as drug carriers, studying the underlying mass transfer mechanisms, and deconvolving a set of relaxation times from the drug release curves. Complicated kinetics of the phase transport restraint at the molecular level can be explained within a simple framework, and the results are indicative for solving practical problems in the design of drug carriers at the engineering level [138,139].

Several factors influence the kinetics of drug release from a delivery system, such as the dose, the dosage form (regardless of the polymer or matrix used), the solubility of the drug, the drug loading capacity, and its release profile. These parameters greatly influence how a drug behaves in the body over time. First-order models are often used to describe the dissolution properties of a drug from a solid dosage form and, to a lesser extent, the dissolution of a drug encapsulated in a polymer matrix. However, if one wishes to use a drug delivery system to modulate the original pharmacokinetic profile, other release kinetics such as zero-order, first-order, or Korsmeyer–Peppas should be considered. Recently, extensive research focus has been placed on the development of analytical methods that incorporate in vitro data that can connect to in vivo projections from polymer devices. Depending on the kinetic model used, different results are observed regarding polymer concentrations at the delivery site [140].

## 5. Applications of PLGA Hydrogels in Drug Delivery

**Figure 7:**
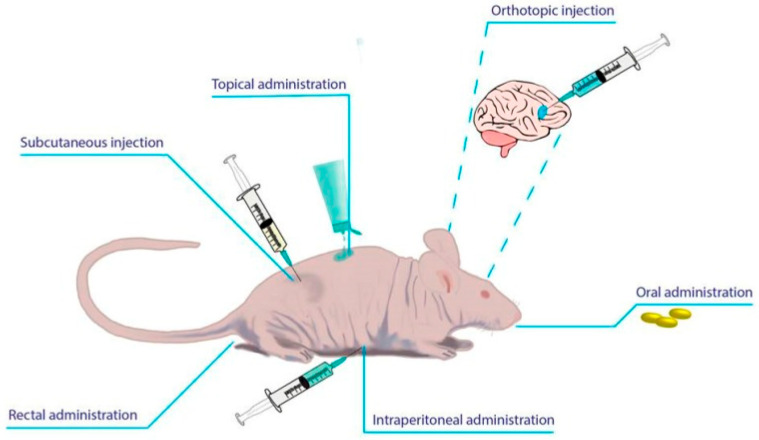
In vivo drug delivery routes of hydrogel administration. There are various methods for in vivo administration of hydrogel-based products depending on the pathological conditions and their localization. Subcutaneous injection plays a crucial role in toxicological effects’ evaluation. Topical or transdermal application is preferred for skin-associated problems. Orthotopic and intraperitoneal injections are non-invasive techniques that ensure good therapy results. On the other hand, oral administration has some limitations because of digestive enzymes [144].

In vivo, drug delivery via hydrogel-based systems encompasses a diverse array of administration routes tailored to specific pathological conditions and anatomical sites [141]. Subcutaneous injection is notable for its pivotal role in evaluating toxicological effects, offering direct access to underlying tissues while facilitating controlled release of therapeutic agents. For skin-related issues, topical or transdermal application proves advantageous, providing localized treatment and bypassing systemic metabolism [142]. Orthotopic and intraperitoneal injections represent non-invasive alternatives that ensure effective therapeutic results depending on the target organ or cavity [31]. However, problems remain with oral administration due to enzymatic degradation in the gastrointestinal tract, limiting its efficacy in delivering hydrogel-based formulations for systemic treatments [143]. Each route of administration offers unique advantages, crucial for optimizing the efficacy and safety of hydrogel-mediated drug delivery systems in clinical applications (Figure 7).

### 5.1. Cancer Therapeutics

Sustained release of PLGA hydrogel formulations is designed to achieve the desired pharmacokinetic profile suitable for localized drug delivery. PLGA, being the most widely used biodegradable polymer in the field of drug delivery, offers the advantage of modulating the release kinetics by tweaking the physicochemical properties, molecular weight, and blend ratio with other biodegradable polymers. Rationally designed PLGA nanoparticles and microspheres integrated within the PLGA hydrogel achieve the sustained release profile. Furthermore, it is shown that the gel matrix can afford synergistic release kinetics. The presence of nanoparticles and microspheres that can hold significant amounts of a drug allows the hydrogel to maintain the initial drug release rates, even for loaded amounts that are potentially higher than what can be offered within the hydrogel itself [145].

Sustained release of chemotherapeutic drugs is an attractive concept in cancer treatment. Certain FDA-approved drugs commonly used in chemotherapy have narrow therapeutic windows where small doses can be therapeutically effective, but higher doses may result in serious side effects. A popular route for localized drug delivery for cancer treatment is through intratumoral injection, where the drug directly enters the tumor. However, intratumoral injection of drugs does away with the dose-sparing effect obtained by using other routes, since a significant amount of the drug is locally or regionally injected. Therefore, a controlled release formulation for local injection can minimize the frequency of drug injections, avoiding declines between repeated injections while maintaining the therapeutic level at the target site for an extended period of time. In the case of localized drug delivery, the requirement of indefinite or chronic expression of the drug to maintain a pharmacological effect essentially translates to the sustained release of the drug [146].

There have been innovations from chemical medicines to immunosuppressive agents and pro-drugs. Use of sustained release is the most significant strategy to extend the block time at the tumor and ensure the least side effects from chemotherapy. Therefore, the innovation should focus on developing various PLGA hydrogels in order to reach comprehensive effects in chemical gene combination chemotherapy based on self-assembly. The hydrogel shell disappears at the moment of contact with apoptosis bodies and exerts excellent properties. Moreover, controllable side-drug release can avoid damage to normal cells. Additionally, controlled release protects the drug in the body by reducing repairs in patients. However, the PLGA hydrogel limits the clinical efficacy because of unsatisfied drug release kinetics for different diseases. Therefore, clinical and global academic innovations are required for various autoimmune diseases [147,148].

Cancer therapeutics are the most developed application of PLGA hydrogels in clinical trials and commercial products. Doxil (Doerr^®^), which is a rapid slow release of doxorubicin, has been commercially available since 1995. A self-assembled PLGA hydrogel is another delivery platform for the rapid and sustained release of doxorubicin [149]. PLGA-based formulations have shown significant cancer therapeutics potential. In many clinical trials for camptothecin, doxorubicin, paclitaxel, and hydroxycamptothecin intravenous injections, most patients achieved CR (complete remission) or PR (partial remission) among the patients in the effective evaluation group, and the symptoms in the control of the distant tumor were obviously inhibited by the sustained release effect of the drug. The 5-Fu hydrogel implant can prolong the release of the drug, and the blood concentration of the drug reaches a high concentration under continuous action, inhibiting the DNA and RNA metabolism of tumor cells. At the same time, GHCR has achieved great success in the prevention of liver cancer and breast cancer using liquid embolization of polymerized drugs (LEPD) and gel micelles [150].

### 5.2. Antibiotics and Antimicrobials

Meanwhile, the increasing growth of antibiotic resistance has become an urgent issue and weakens the therapeutic efficacy of antibiotics, leading to a high financial burden and increased mortality, competing with cancer. The question arises of what to do to combat drug resistance and launch the continuous production of new antibiotics with novel mechanisms [151]. In response, 3D printing, known for its high versatility in producing 3D structures with designed architectures, has been widely used to produce 3D scaffolds for bacterial purification and drug release of various antibiotics. In addition, it can be directly integrated with filament formulations of drugs as well as metal oxide nanoparticles to fabricate antibacterial materials by hybrid methods. This technology can not only provide high flexibility for fabricating complex and customized structures but also enable region-controlled drug release and site-specific treatment [152].

PLGA hydrogels are promising for localized drug release, and their development has been moving toward an advanced stage. Over the past decade, hydrogels made with PLGA and polyethylene glycol (PEG) have been extensively developed for the sustained delivery of antibiotics and antimicrobials. These hydrogels have demonstrated excellent performance for anti-infection in vivo. A number of antibiotics, ranging from small molecules like ofloxacin, ciprofloxacin, and gentamicin to large proteins like LL-37, have been successfully encapsulated in PLGA hydrogel and released for an extended period without any loss of original activity. More importantly, through optimized formulations or post-synthesis treatment, the hydrogels do not cause any harm or side effects to the local cells during drug release, both in vitro and in vivo [153].

In skeletal tissue engineering and spinal fusion, PLGA hydrogel has been extensively studied, primarily due to its bone tissue compatibility. A study used PLGA hydrogel to encapsulate gentamicin and reported that PLGA hydrogels exhibited an ability to control gentamicin release and exhibited sustained release over a 56-day study at concentrations remaining above the bacteria sensitivity level. Another study investigated a PLGA hydrogel containing vancomycin and studied the effects of antimicrobial activity and degradation-related cytotoxicity. These results suggest a potential use for PLGA hydrogel in clinical applications requiring localized release of antibiotics at a minimal repeated dose for the prevention of surgical site infections [154,155]. 

As a class of persistent clinical problems, hardware-associated infection (HAI) is caused by bacterial colonization and biofilm formation on the surface of surgical implants, such as plates, screws, and balance nails, which are often used directly or temporarily in the clinic [156]. For the prevention and treatment of HAI, the local administration of high-concentration antibiotics plays an important role because the systemic administration of antibiotics not only cannot achieve an efficient therapeutic concentration but also results in inevitable side effects. Therefore, a variety of polymers, metal nanoparticles, and fibers have been used to load antibiotics to prepare local antibiotic delivery systems. The high-density pores in hydrogels can provide a reserve for loading growth factors and antibiotics, while the water content satisfies the water environment that is favorable for the activities of transplanted cells [157].

## 6. Challenges and Future Perspectives in PLGA Hydrogel Research

This section presents the utilization of PLGA hydrogel-based products in clinical trials aimed at achieving sustained drug release. Table 2 provides a comprehensive summary of various completed clinical trials investigating the efficacy and safety of PLGA hydrogels across different medical applications. By examining these studies, we can gain valuable insights into the potential of PLGA hydrogels to improve treatment outcomes and improve patient compliance.

PLGA has low cost and excellent biocompatibility. Moreover, it has been approved by the Food and Drug Administration (US FDA) for therapeutic medicine application. However, it has a hydrophobic nature and the experimental procedures have required toxic reagents. The adding processes only require mechanical force without damage to cell viability at the mixing or injected points. The synthesized PLGA hydrogel has good biocompatibility [162]. 

Over the last decade, the use of the copolymer poly(l-lactide-co-glycolide) (PLGA) has become widespread, in part due to its versatility and the physical forms that it can be induced to take, such as sutures, pins, screws, films, and microparticles. The desired characteristics of PLGA have been exploited to realize a range of applications that take advantage of its biocompatibility and biodegradability. In addition, hydrogels based on PLGA copolymers have been developed. One of the most versatile polymers currently available in the biomedical field is PLGA, successfully developed for drug therapy, wound healing therapy in the form of adsorbed sheets, and devices in the form of injectable microparticle-based formulations that possess desirable properties [37]. The incorporation of PLGA microparticles into polymeric hydrogels further extends their utility in a broad range of clinical applications and shows this combination to hold the potential to create the ultimate sustained release system. An overview of the development and applications of the PLGA employed as microparticles and the newly developed PLGA hydrogel is given [163]. Biodegradable PLGA hydrogels, in vitro or in vivo, have been applied to collagen, chitosan, gelatin, hyaluronic acid, etc. Usually, based on their own combined self-crosslinking and other crosslinking methods, they are often involved in several analytical methods, including tensile strength, brittleness, biodegradability, adult stem cells, growth factors, enzymes, antibacterial agents, factors with slow release effects, and gene factors. These can be combined to focus on the development of regenerative medicine, cancer treatment, and many other needs [164].

### Enhanced Drug Loading and Release Control

PLGA-based nanoparticles have been used to control the drug release through an inactive bake-cast method. Methoxy poly(ethylene glycol)-b-poly(L-lactide-co-N-2-(N′3-aminopropyl)guanidine-L-aspartamide) (mPEG-b-PLA-co-PLGA) or PE, both linked with the luteinizing hormone releasing hormone peptide (LHRH), were incorporated into nanoparticles. Liver tissue differences influenced the safety and anti-tumor efficiency of PLGA–Doxin–LHRH nanoparticles [165].

The ophthalmic drug release and biocompatibility of secnidazole PLGA nanoparticles were tested. Other authors observed that an addition of cyclodextrins can release the in vivo model drug encapsulated by PLGA nanoparticles. The developments should improve the effectiveness and therapeutic index [166].

PLGA hydrogels made by dialysis and low temperature can increase encapsulation rates (or loading rates) to a certain extent. Their sol-gel phase transition occurs at low temperature, which does not damage the encapsulated proteins. A PLGA hydrogel prepared at the controlled release stage showed more sustained delivery profiles, even after the introduction of the fresh active ingredient. The increased therapeutic agent release module was physically trapped in the PLGA hydrogel. H. Chen et al. indicated that the PLGA–Porex hydrogel is also effective in maintaining drug release during multiple dosing systems [167]. The porosity control of hydrogel can effectively inhibit drug burst release and greatly control the uptake capacity [168].

## 7. Conclusions

PLGA hydrogels have emerged as a highly versatile and effective platform for the sustained delivery of therapeutic agents. Their adjustable biodegradability, biocompatibility, and mechanical properties make them suitable for a wide range of biomedical applications. Through their controlled synthesis and strategic modification, PLGA hydrogels can be engineered to provide precise drug release profiles, addressing the unique requirements of various therapeutic scenarios. The successful application of PLGA hydrogels in drug delivery systems for diseases such as cancer, diabetes, and tissue regeneration highlights their potential to significantly improve treatment efficacy and patient compliance.

Despite these promising advancements, several challenges remain, including optimizing drug loading and release kinetics, scaling up production processes, and ensuring consistent performance in clinical settings. Future research should focus on overcoming these obstacles, exploring novel modifications, and integrating advanced technologies such as nanotechnology and personalized medicine approaches. Overall, PLGA hydrogels represent a robust and adaptable platform with the potential to revolutionize the sustained delivery of therapeutic agents and open new horizons in medical treatments and patient care.

## Figures and Tables

**Table 1:** Overview of Different Synthesis Methods for PLGA Hydrogels.

Synthesis Method	Advantages	Disadvantages	Case Example (Drug Released)	Ref.
Emulsion Solvent Evaporation	Good control over particle size; high drug loading	Requires use of organic solvents	Dexamethasone	[41]
Solvent Casting/Particulate Leaching	Creates porous structure; simple method	Potential residual solvents; limited to small-scale production	Vancomycin	[46]
Electrospinning	High surface area; controlled release profile	Complex setup; requires high voltage	Ciprofloxacin	[47]
Thermal Gelation	No need for organic solvents; temperature-controlled gelation	Limited to thermosensitive drugs; potential thermal degradation	BSA (Bovine Serum Albumin)	[48]
Photopolymerization	Precise control over gelation; spatial control	Requires photoinitiators; potential UV damage to drugs	Methotrexate	[49]

**Table 2:** Summary of Commercial PLGA-Based Products.

Commercial Product	Drug Release in PLGA Matrix	Benefit	Reference
Lupron Depot	Leuprolide acetate released over 1–6 months	Treats prostate cancer, endometriosis, and uterine fibroids with sustained release	[158]
Zoladex	Goserelin acetate released over 1–3 months	Provides hormone therapy for prostate and breast cancer with controlled release	[159]
Sandostatin LAR	Octreotide acetate released over 1 month	Treats acromegaly and severe diarrhea by sustained drug levels	[160]
Risperdal Consta	Risperidone released over 2 weeks	Treats schizophrenia with prolonged therapeutic effect	[161]

## Data Availability

Not applicable.
